# Impact of l‐citrulline on nitric oxide signaling and arginase activity in hypoxic human pulmonary artery endothelial cells

**DOI:** 10.1002/pul2.12221

**Published:** 2023-04-01

**Authors:** Matthew S. Douglass, Mark R. Kaplowitz, Yongmei Zhang, Candice D. Fike

**Affiliations:** ^1^ Department of Pediatrics University of Utah Salt Lake City Utah USA

**Keywords:** neonate, newborn, pulmonary hypertension, pulmonology

## Abstract

Impaired nitric oxide (NO) signaling contributes to the development of pulmonary hypertension (PH). The l‐arginine precursor, l‐citrulline, improves NO signaling and has therapeutic potential in PH. However, there is evidence that l‐citrulline might increase arginase activity, which in turn, has been shown to contribute to PH. Our major purpose was to determine if l‐citrulline increases arginase activity in hypoxic human pulmonary artery endothelial cells (PAECs). In addition, to avoid potential adverse effects from high dose l‐citrulline monotherapy, we evaluated whether the effect on NO signaling is greater using co‐treatment with l‐citrulline and another agent that improves NO signaling, folic acid, than either alone. Arginase activity was measured in human PAECs cultured under hypoxic conditions in the presence of l‐citrulline (0–1 mM). NO production and endothelial nitric oxide synthase (eNOS) coupling, as assessed by eNOS dimer‐to‐monomer ratios, were measured in PAECs treated with l‐citrulline and/or folic acid (0.2 μM). Arginase activity increased in hypoxic PAECs treated with 1 mM but not with either 0.05 or 0.1 mM l‐citrulline. Co‐treatment with folic acid and 0.1 mM l‐citrulline increased NO production and eNOS dimer‐to‐monomer ratios more than treatment with either alone. The potential to increase arginase activity suggests that there might be plasma l‐citrulline concentrations that should not be exceeded when using l‐citrulline to treat PH. Rather than progressively increasing the dose of l‐citrulline as a monotherapy, co‐therapy with l‐citrulline and folic acid merits consideration, due to the possibility of achieving efficacy at lower doses and minimizing side effects.

## INTRODUCTION

Dysregulation of the nitric oxide (NO) pathway plays a role in the pathobiology of pulmonary hypertension (PH) in humans.^1^, ^2^ Studies performed in animal models provide preclinical evidence that targeting the NO pathway can be an effective way to treat PH.^3^, ^4^, ^5^, ^6^, ^7^ For example, in studies using a chronically hypoxic newborn piglet animal model, we found that treatment with the l‐arginine‐NO precursor, l‐citrulline concomitantly increases NO production and inhibits the development of PH in a dose‐dependent fashion.^8^, ^9^

Our findings in chronically hypoxic piglets might suggest that continuing to increase the dose of l‐citrulline should lead to progressive improvements in the degree of PH found in this animal model. However, findings from studies showing that higher doses of l‐citrulline increase arginase expression and activity in hypoxic piglet pulmonary artery endothelial cells (PAECs), indicates that l‐citrulline dosing should be increased with caution. Arginase can have a detrimental effect on NO signaling by competing with endothelial nitric oxide synthase (eNOS) for the substrate, l‐arginine.^10^, ^11^ Furthermore, arginase has been purported to contribute to a variety of cardiovascular diseases, including PH, in part because of its negative impact on NO production.^12^, ^13^, ^14^

Our ultimate goal is to translate our findings from our animal model to clinical studies designed to evaluate l‐citrulline as a therapeutic intervention for PH in humans. When designing clinical trials in humans, the potential for adverse effects from the therapeutic intervention must always be considered. The major purpose of this study was therefore to determine whether supplemental l‐citrulline induces arginase expression and activity in hypoxic human PAECs as we found it does in hypoxic piglet PAECs. Moreover, we reasoned that to avoid the potential to uncover adverse consequences from using high‐dose monotherapy, combining l‐citrulline with another agent that positively impacts NO signaling might be a useful therapeutic strategy. The water‐soluble B vitamin, folic acid, has been shown to improve NO signaling.^15^ Therefore, we also performed studies to determine the effect of combining l‐citrulline with folic acid on NO signaling in hypoxic human PAECs.

## METHODS

Human PAECs (Lonza) were cultured in endothelial cell growth medium (EGM; Lonza) in a humidified normoxic incubator at 37°C until approximately 70% confluence. The nearly confluent PAECs were subcultured at 37°C in a humidified environment under hypoxic conditions, 4% oxygen and 5% carbon dioxide, for 48 h. Hypoxic PAECs were treated with either 0 mM l‐citrulline, 0.05 mM l‐citrulline, 0.1 mM l‐citrulline, or 1.0 mM l‐citrulline (Sigma‐Aldrich). Additional hypoxic PAECs were cultured in the presence and absence of l‐citrulline (0.1 mM) or folic acid (0.2 μM), or were co‐treated with l‐citrulline (0.1 mM) and folic acid (0.2 μM).

Hypoxic PAECs were used for immunoblot analyses. After culturing for 48 h under hypoxic conditions as described above, PAECs were washed with phosphate‐buffered saline (PBS), then covered with cell lysis buffer (Thermo Fisher Scientific), protease inhibitor cocktail (Sigma‐Aldrich), and phenylmethylsulfonyl fluoride (Sigma‐Aldrich). Following centrifugation, aliquots of supernatant were collected and stored at −80°C. For use in the immunoblot analyses, stored aliquots of frozen supernatant were used for protein concentration by Bradford assay. For arginase I and II and eNOS analysis, using previously described methods, aliquots of supernatant were applied to tris‐glycine precast 4%–20% polyacrylamide gels so that equal amounts of protein were loaded.^16^ After electrophoresis at 120 volts for 2 h, the protein was transferred to a polyvinylidene difluoride membrane. Nonspecific protein binding was blocked by incubating the membrane at room temperature in PBS and 0.1% Tween‐20 (PBS‐T; Sigma‐Aldrich) containing 5% nonfat dried milk and then washing in PBS‐T containing 1% nonfat dried milk. The protein of interest was detected by incubating the membrane overnight with arginase I (Abcam) 1:500, arginase II (Santa Cruz Biotechnology) 1:500, or eNOS (BD Transduction Laboratories) 1:1000. All antibodies were diluted in PBS containing 0.1% Tween‐20% and 1% nonfat dried milk as carrier buffer. Next, the membrane was washed with PBS‐T and 1% nonfat dried milk, then incubated for 60 min with anti‐rabbit (for arginase I and II, Cell Signaling Technology) or anti‐mouse (for eNOS; Cell Signaling Technology) IgG horseradish peroxidase (HRP)‐linked secondary antibody diluted in the carrier buffer (1:2500). Using previously described methods, nonsonicated and nonboiled lysates and low‐temperature SDS‐PAGE were used for eNOS dimers and monomers.^10^, ^16^, ^17^ Enhanced chemiluminescence reagents (PerkinElmer) were used to develop the membranes and an iBright FL1500 imaging system (Thermo Fisher Scientific) was used to capture the bands. The bands for each protein were quantified using the iBright Analysis Software. Then the membranes were washed and stripped and re‐probed for beta‐actin (1:100,000).

Using previously described methods, arginase activity was measured in PAECs that had been cultured under hypoxic conditions for 48 h as described above under PAEC Experimental Protocols.^16^ Briefly, an arginase assay kit (Abnova) was used to measure arginase activity by colorimetric determination of urea produced by arginase. Protein content was determined by Bradford assay and used to normalize arginase activity.

Previously described methods were used to measure NO concentrations for hypoxic PAECs.^16^ For these studies, PAECs were cultured under hypoxic conditions for 48 h as described above under PAEC Experimental Protocols. After washing with PBS, the PAECs were then incubated under hypoxic conditions for an additional 60 min in Krebs–Hepes buffer with A‐23187 (10 mM), l‐arginine (10 mM), and the same concentrations of l‐citrulline, folic acid, or combination of l‐citrulline and folic acid, as used for the initial 48 h incubation. The Ca^2+^ ionophore A23187 is added to the cultured PAECs to promote dissociation of eNOS from caveolin, a step necessary for activation of eNOS' enzymatic activity.^17^ Aliquots of the Krebs–Hepes buffer were injected into the reaction chamber of a chemiluminescent NO analyzer (Sievers). The reaction chamber contained vanadium (III) chloride in 1 M HCl at 90°C to reduce nitrite and nitrate to NO gas. N_2_ gas was used to transfer the NO gas to a gas bubble trap where HCl vapor was removed by 1 M NaOH. NO concentrations from the human PAECs were determined using a standard curve generated from NaNO_3_ concentrations of 1, 2.5, 5, 10, 15, and 20 μM. The NO concentration was normalized to protein content which was determined by Bradford assay.

Using previously described methods, superoxide (O_2_^−^) was measured in hypoxic PAECs by lucigenin (N,N′‐Dimethyl‐9.9′‐biacridinium dinitrate) enhanced chemiluminescence.^16^ First, PAECs were cultured under hypoxic conditions for 48 h as described above under PAEC Experimental Protocols. After washing with PBS, the PAECs were then incubated under hypoxic conditions for an additional 60 min in Krebs Hepes buffer with A‐23187 (10 mM; Biomol), l‐arginine (10 mM; Sigma‐Aldrich), and the same concentrations of l‐citrulline, folic acid, or combination of l‐citrulline and folic acid as used for the initial 48 h incubation. Next, the PAECs were placed into the luminometer (Titertek Instruments) and background relative light units (RLU) were measured. Lucigenin (5 mM) was then added and lucigenin‐treated RLU measurements obtained. The background RLU measurements were subtracted from lucigenin‐treated RLU measurements and normalized to protein content which was determined by Bradford assay.

Data are presented as mean ± SD. One‐way analysis of variance with Fisher's protected least significant differences post hoc comparison test was used to compare data between groups. A *p* < 0.05 was considered significant.^18^

## RESULTS

**Figure 1:**
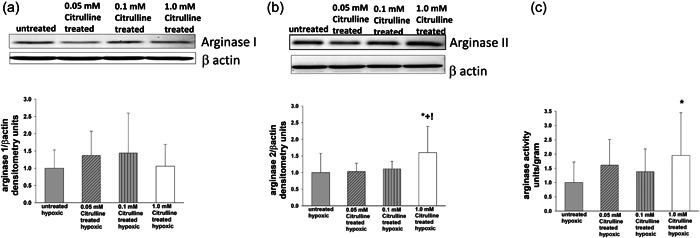
Arginase I and II protein levels and arginase activity in hypoxic human PAECs treated with and without l‐citrulline. (a) Arginase I protein levels were similar between untreated and l‐citrulline treated hypoxic pulmonary artery endothelial cells (PAECs) regardless of concentration of l‐citrulline (*n* = 12 for each group). (b) Arginase II protein levels were greater for hypoxic PAECs treated with 1.0 mM l‐citrulline compared with any of the other three groups (*n* = 12 for each group, *p < 0.05 from hypoxic untreated, !*p* < 0.05 from hypoxic treated with 0.05 mM l‐citrulline, +*p* < 0.05 from hypoxic treated with 0.1 mM l‐citrulline). (c) Arginase activity was greater for hypoxic PAECs treated with 1.0 mM l‐citrulline than for untreated hypoxic PAECs. (*n* = 16 for each group, *p < 0.05 from hypoxic untreated).

The impact of l‐citrulline treatment on arginase I and II protein amounts and arginase activity in hypoxic human PAECs was evaluated in the first series of studies. Arginase I protein amounts were similar for untreated and l‐citrulline‐treated hypoxic human PAECs regardless of l‐citrulline concentration (Figure 1a). Arginase II protein amounts did not differ between untreated hypoxic PAECs and those treated with either of the two lower concentrations of l‐citrulline (0.05 or 0.1 mM l‐citrulline) (Figure 1b). However, arginase II protein amounts were greater for hypoxic PAECs treated with the highest l‐citrulline concentration (1.0 mM) than for any of the other three groups (Figure 1b). Arginase activity did not differ between untreated hypoxic PAECs and hypoxic PAECs treated with either of the two lower l‐citrulline concentrations (0.05 or 0.1 mM). Notably, arginase activity was greater for hypoxic PAECs treated with the highest l‐citrulline concentration (1 mM) than for untreated hypoxic PAECs (Figure 1c). Thus, treatment with 1 mM l‐citrulline increased both arginase activity and arginase II protein levels in hypoxic human PAECs.

**Figure 2:**
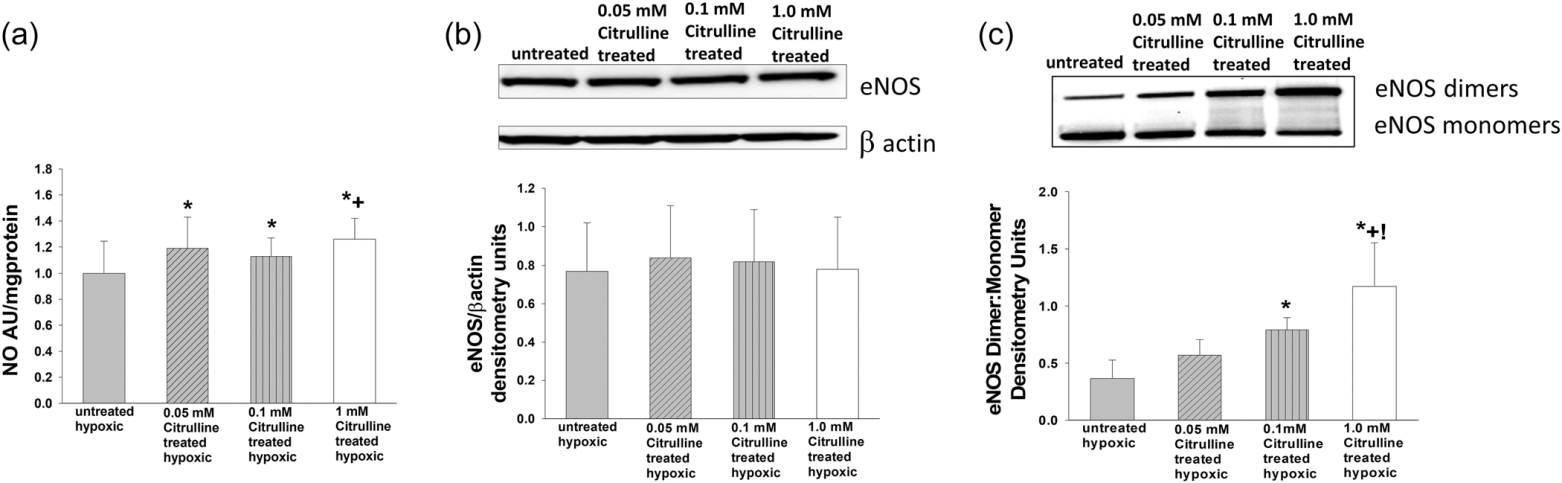
NO production, total eNOS protein levels, and eNOS dimer to monomer ratios in hypoxic PAECs treated with and without l‐citrulline. (a) Nitric oxide (NO) production was greater for all groups of l‐citrulline treated hypoxic pulmonary artery endothelial cells (PAECs) than for untreated hypoxic PAECs. In addition, NO production was greater for hypoxic PAECs treated with 1.0 mM l‐citrulline compared to 0.1 mM l‐citrulline (*n* = 15 for each group, *p < 0.05 from hypoxic untreated, +*p* < 0.05 from 0.1 mM l‐citrulline). (b) There was no difference in total endothelial nitric oxide synthase (eNOS) protein levels amongst the groups of hypoxic PAECs (*n* = 6 for each group). (c) ENOS dimer‐to‐monomer ratios were greater for hypoxic PAECs treated with either 0.1 mM or 1.0 mM l‐citrulline than for untreated hypoxic PAECs. In addition, eNOS dimer‐to‐monomer ratios were greater for hypoxic PAECs treated with 1.0 mM l‐citrulline than for all other groups. (*n* = 6 for each group, *p < 0.05 from hypoxic untreated, !*p* < 0.05 from hypoxic 0.05 mM l‐citrulline, +*p* < 0.05 from hypoxic 0.1 mM l‐citrulline).

We then evaluated the effect of l‐citrulline concentration on NO signaling in hypoxic human PAECs. For these studies we measured the impact of l‐citrulline concentration on NO production, total eNOS protein amounts, and the state of eNOS coupling, as reflected by eNOS dimer‐to‐monomer ratios. Compared with the untreated group, NO production was higher for hypoxic PAECs treated with any of the three concentrations of l‐citrulline (Figure 2a). Moreover, NO production was greater for those hypoxic PAECs treated with the highest concentration of l‐citrulline (1.0 mM) than for those treated with the middle concentration of l‐citrulline (0.1 mM) (Figure 2a). Thus, l‐citrulline increased NO production in hypoxic human PAECs (Figure 2a) even when treated with a concentration of l‐citrulline (1.0 mM) that induced arginase activity (Figure 1c).

Total eNOS amounts were similar for all groups of hypoxic PAECs, regardless of the presence or absence of any concentration of l‐citrulline (Figure 2b). Both the middle (0.1 mM) and highest (1.0 mM) concentrations of l‐citrulline increased eNOS dimer‐to‐monomer ratios in hypoxic human PAECs above the ratio found in untreated hypoxic PAECs (Figure 2c). Moreover, eNOS dimer‐to monomer ratios were greater for hypoxic human PAECs treated with the highest concentration of l‐citrulline than the ratios measured in any of the other three groups (Figure 2c). This finding indicates that despite treatment with a concentration of l‐citrulline that induced arginase activity (Figure 1c), l‐citrulline re‐coupled eNOS in hypoxic human PAECs (Figure 2c).

**Figure 3:**
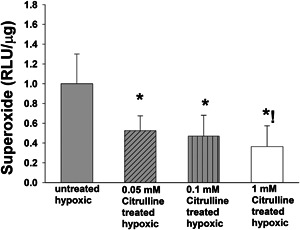
O_2_.^−^ generation in hypoxic PAECs treated with and without l‐citrulline. Superoxide (O_2_.^−^) generation was less for all groups of l‐citrulline treated hypoxic pulmonary artery endothelial cells (PAECs) than for untreated hypoxic PAECs. In addition, O_2_.^−^ generation was less for hypoxic PAECs treated with 1.0 mM l‐citrulline compared to 0.05 mM l‐citrulline (*n* = 10 for each group, *p < 0.05 from hypoxic untreated, !*p* < 0.05 from 0.05 mM l‐citrulline).

We also evaluated the impact of l‐citrulline concentration on O_2_^−^ production. All concentrations of l‐citrulline reduced O_2_^−^ production in hypoxic human PAECs to levels below those measured in the untreated group (Figure 3). Furthermore, the amount of O_2_^−^ generated by hypoxic PAECs treated with the highest concentration of l‐citrulline (1 mM) was less than the amount generated by those treated with the lowest concentration of l‐citrulline (0.05 mM) (Figure 3). Thus, l‐citrulline reduced O_2_^−^ generation in hypoxic human PAECs (Figure 3) even when treated with a concentration found to increase arginase activity (Figure 1c).

**Figure 4:**
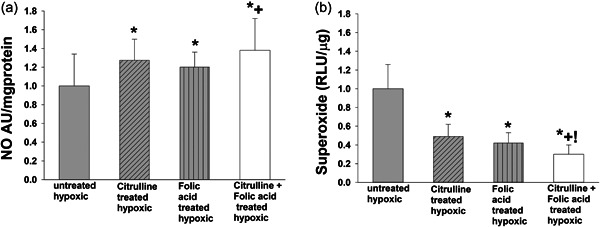
NO production and O_2_.^−^ generation in hypoxic PAECs treated with l‐citrulline and/or folic acid. (a) Regardless of treatment strategy, nitric oxide (NO) production was greater for all three groups of treated hypoxic pulmonary artery endothelial cells (PAECs) compared to untreated hypoxic PAECs. There was no significant difference in NO production between PAECs treated solely with either l‐citrulline or folic acid. NO production was greater for hypoxic PAECs co‐treated with l‐citrulline and folic acid compared to hypoxic PAECs treated solely with folic acid (*n* = 13, *p < 0.05 from hypoxic untreated, +*p* < 0.05 for sole treatment with folic acid). (b) Regardless of treatment strategy, superoxide (O_2_^−^) generation was less for all three groups of treated hypoxic PAECs compared to untreated hypoxic PAECs. The amount of O_2_^−^ generated did not differ between PAECs treated solely with either l‐citrulline or folic acid. However, the amount of O_2_^−^ generated was less for hypoxic PAECs co‐treated with l‐citrulline and folic acid than for hypoxic PAECs treated with either l‐citrulline or folic acid alone. (*n* = 10 for each group, *p < 0.05 from hypoxic untreated, +*p* < 0.05 from sole treatment with folic acid, !*p* < 0.05 from sole treatment with l‐citrulline).

**Figure 5:**
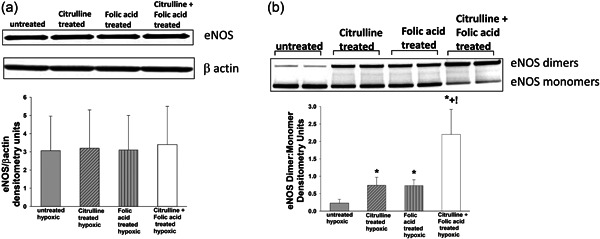
Total eNOS protein levels and eNOS dimer to monomer ratios in hypoxic PAECs treated with l‐citrulline and/or folic acid. (a) There was no difference in total eNOS protein levels amongst the groups of hypoxic PAECs (*n* = 5 for each group). (b) Regardless of treatment strategy, endothelial nitric oxide synthase (eNOS) dimer‐to‐monomer ratios were greater for all three groups of treated hypoxic PAECs compared to untreated hypoxic PAECs. eNOS dimer‐to‐monomer ratios did not differ between PAECs treated solely with either l‐citrulline or folic acid. However, eNOS dimer‐to‐monomer ratios were greater for hypoxic PAECs co‐treated with l‐citrulline and folic acid than for hypoxic PAECs treated with either l‐citrulline or folic acid alone. (*n* = 10 for each group, *p < 0.05 from hypoxic untreated, +*p* < 0.05 from sole treatment with folic acid, !*p* < 0.05 from sole treatment with l‐citrulline).

Based on the desire to explore alternative therapeutic approaches to high‐dose l‐citrulline monotherapy, we then evaluated the impact of combined treatment with l‐citrulline and folic acid versus sole treatment with l‐citrulline or folic acid on NO production, eNOS coupling, and O_2_^−^ generation in hypoxic human PAECs. Levels of NO produced by hypoxic human PAECs treated either solely with l‐citrulline, solely with folic acid, or co‐treated with l‐citrulline and folic acid were greater than levels of NO produced by untreated hypoxic PAECs (Figure 4a). Moreover, NO levels produced by hypoxic human PAECs co‐treated with l‐citrulline and folic acid were higher than NO levels produced by hypoxic PAECs treated solely with folic acid (Figure 4a). Regardless of treatment strategy, the amount of O_2_^−^ generated by all three treated groups of hypoxic PAECs was less than the amount of O_2_^−^ generated by the untreated group (Figure 4b). Lower levels of O_2_^−^ were generated by hypoxic PAECs co‐treated with both l‐citrulline and folic acid than by hypoxic PAECS treated with either l‐citrulline or folic acid alone (Figure 4b). Total eNOS amounts were similar for untreated and treated hypoxic human PAECs (Figure 5a). ENOS dimer‐to‐monomer ratios were lower for the untreated group of hypoxic human PAECs than for any of the three treated groups (Figure 5b). Of note, eNOS dimer‐to‐monomer ratios were higher for hypoxic human PAECS co‐treated with l‐citrulline and folic acid than for hypoxic PAECs treated with either l‐citrulline or folic acid alone.

## DISCUSSION

In this study, we found that 1.0 mM l‐citrulline increases arginase II expression and arginase activity in hypoxic human PAECs. These results are consistent with findings in our previous study in hypoxic newborn piglet PAECs.^16^ The impact of hypoxia on arginase and other aspects of the NO signaling pathway have been noted to differ between species; thus, it was important to investigate if l‐citrulline also regulates arginase expression and activity in human PAECs as there has been increased interest in l‐citrulline as a treatment for PH in humans.^19^, ^20^, ^21^ Determining the optimal plasma l‐citrulline level that produces a therapeutic response while avoiding unintended side effects, such as increasing arginase expression and activity, will be important for developing future clinical trials evaluating the safety and efficacy of l‐citrulline for treating PH.

In the present study, we drew upon our prior experience studying l‐citrulline in piglets to select concentrations of l‐citrulline that mimic plasma levels that would be appropriate to target therapeutically in humans. In these piglets we found that trough plasma l‐citrulline levels that were at least 50% above basal plasma levels were associated with a dose‐dependent amelioration of chronic hypoxia‐induced PH.^8^, ^9^ Thus, based on the magnitude of increase in plasma l‐citrulline concentrations that produced therapeutic efficacy in piglets, one reasonable strategy for a future clinical trial in human infants and children with PH would be to evaluate the effect of l‐citrulline therapy when plasma l‐citrulline levels are achieved that are at least 50% above basal levels. Published basal plasma l‐citrulline levels in human infants and children are 0.013–0.069 mM.^22^ Further, in an l‐citrulline pharmacokinetics study preterm infants at risk of bronchopulmonary dysplasia associated pulmonary hypertension were found to have a median basal l‐citrulline concentration of 31 µmol/L (0.031 mM) with a range of 9–36 µmol/L (0.009–0.036 mM).^23^ The targeted steady‐state of plasma l‐citrulline concentration in this pharmacokinetics study was 50–80 µmol/L (0.05–0.08 mM) which reflects at least a 50%–100% increase from median basal plasma l‐citrulline concentrations in the study participants. However, it merits comment that other investigators studying l‐citrulline and pulmonary hypertension in children have targeted slightly higher plasma l‐citrulline concentrations (80–100 µmol/L).^24^ Therefore, in this study, we aimed to determine whether arginase expression and activity are increased in hypoxic human PAECs exposed to l‐citrulline concentrations of 0.05–1.0 mM, a range that includes concentrations greater than 50% above basal l‐citrulline levels.

Of great interest, we found that arginase II amounts and arginase activity were increased when hypoxic human PAEC were exposed to 1 mM l‐citrulline, but not when they were exposed to 0.05 or 0.1 mM concentrations of l‐citrulline. In other words, our findings provide some reassurance that targeting plasma l‐citrulline concentrations up to 0.1 mM, a concentration greater than 50% basal concentration in human infants and children, will not increase arginase expression or activity in vivo. These findings are clinically relevant because arginase has been implicated in the pathogenesis of a number of cardiovascular diseases, including PH.^11^, ^12^, ^13^, ^14^, ^25^

Inconsistent with our findings, not all investigators have provided evidence that l‐citrulline induces arginase expression or activity.^26^, ^27^ In fact, one group of investigators reported that patients with type 2 diabetes had a 21% reduction in plasma arginase activity following treatment with l‐citrulline for 1 month.^26^ The same group of investigators performed studies with bovine aortic endothelial cells and found that treatment with l‐citrulline inhibited high glucose‐induced elevations in arginase activity.^26^ Another group of investigators found that l‐citrulline reversed hyperoxia‐induced elevations in arginase activity in lung tissue from a newborn rat model of bronchopulmonary dysplasia.^27^ It is possible that the impact of l‐citrulline on arginase regulation will differ dependent on species, animal ages, vascular bed, cell type, and disease condition being treated.

Our findings in both this and our previous study suggest that regulation of arginase by l‐citrulline is isoform‐dependent. Specifically, although both known isoforms of arginase, arginase I and arginase II, were demonstrated to be present in hypoxic human PAECs in this study and in hypoxic piglet PAECs in our previous study, arginase II protein levels but not arginase I protein levels, were increased with l‐citrulline supplementation in hypoxic PAECs from both species.^16^ This finding is not surprising since it is known that arginase I and arginase II are encoded by separate genes and are known to be regulated by different stimuli.^11^, ^25^ For example, other investigators have found that hypoxia upregulates arginase II but not arginase I in human pulmonary microvascular endothelial cells and further that the upregulation was oxygen concentration dependent.^20^, ^28^ The contribution of hypoxia on l‐citrulline induced arginase activity in our studies is unclear. It is possible that the hypoxic conditions alone contribute to increased arginase activity and l‐citrulline may exacerbate that increased activity. Future studies could include human PAECs treated with l‐citrulline and cultured under both normoxic and hypoxic conditions with and without the presence of an arginase inhibitor such as S‐(2‐borono‐ethyl)‐l‐cysteine (BEC) to provide additional insight on the regulation of arginase by l‐citrulline in human PAECs.

The potential therapeutic impact from the differential effect of l‐citrulline on arginase II and arginase I expression merits further comment. There is a growing appreciation that in addition to modulating NO production by endothelial cells, both arginase I and arginase II may play important roles in regulating a variety of functions in a number of other cell types.^29^ For example, arginase II expression has been shown to trigger macrophage proinflammatory responses and potentially contribute to the development of some vascular diseases including atherosclerosis.^30^ Yet, upregulation of arginase I has been shown to decrease macrophage infiltration and inflammation in atherosclerotic plaques of rabbits which could mitigate the pathological changes that contribute to atherosclerosis.^31^ It is notable that macrophages have been purported to play a complex role in the pathogenesis of pulmonary hypertension by participating in both developmental and reparative stages of the disease process.^32^, ^33^ Thus, it must be considered that treatment with l‐citrulline may have unforeseen and potentially detrimental effects on the pathogenesis of pulmonary hypertension via differential impacts on the role of arginase I and/or arginase II in cell types other than endothelial cells by mechanisms not currently well understood.

It is not surprising that in this study we found that supplementation with l‐citrulline increased NO production, elevated eNOS dimer‐to‐monomer ratios, and reduced superoxide generation in hypoxic human PAECs. These findings are consistent with our previous in vivo findings in chronically hypoxic newborn piglets and with our in vitro findings in hypoxic piglet PAECs.^16^, ^34^ Taken together, our findings continue to indicate that l‐citrulline mechanistically improves eNOS dysfunction in hypoxic conditions by recoupling eNOS.

It is important to note that in both our previous study with hypoxic piglet PAECs and in this study with hypoxic human PAECs, the improvement in eNOS dysfunction occurred even when concentrations of l‐citrulline were used that induced arginase II expression and arginase activity. Moreover, in vivo treatment with l‐citrulline had an ameliorative effect on the development of chronic hypoxia‐induced PH even though some piglets achieved plasma concentrations of l‐citrulline that were found to induce arginase activity in vitro.^16^ However, because arginase is known to modulate NO production by competing with eNOS for the common substrate, l‐arginine, it is possible that an even greater improvement in eNOS function and amelioration of PH in the piglets would have taken place had the induction of arginase not occurred.^11^, ^35^, ^36^

Rather than take a risk to negatively impact eNOS function by increasing arginase activity with l‐citrulline concentrations exceeding 0.1 mM, we reasoned that combining l‐citrulline with another agent that positively impacts NO signaling might be a useful therapeutic strategy to pursue in future human clinical trials. Therefore, we performed studies to evaluate the impact of combining l‐citrulline with folic acid on NO signaling in hypoxic human PAECs because folic acid has been shown to improve NO signaling by a number of mechanisms, including increasing the effectiveness of the eNOS cofactor tetrahydrobiopterin (BH_4_), positively impacting phosphorylation of eNOS, and recoupling eNOS.^4^, ^37^ For these l‐citrulline and folic acid combination studies we selected the l‐citrulline concentration of 0.1 mM based on the published studies outlining l‐citrulline concentrations to target in preterm infants and children while also taking into account our results in this study showing that l‐citrulline at 0.1 mM contributes to the desired therapeutic effects of improving NO bioavailability while potentially minimizing the adverse effect of inducing arginase.^23^, ^24^

In this study, we found that treatment with folic acid alone increased NO production and eNOS dimer‐to‐monomer ratios and reduced superoxide generation. These findings indicate that at least one of the mechanisms by which folic acid improves NO production in hypoxic human PAECs is via improving eNOS coupling.

Another reason underlying our interest in evaluating folic acid is that previous studies by us with chronically hypoxic piglets and by another group of investigators with chronically hypoxic adult mice have shown that folic acid treatment improves PH in chronically hypoxic animal models.^38^ Moreover, although the impact on human pulmonary vascular disease has not been studied, there are a number of publications providing evidence that folic acid can be an effective treatment for a variety of cardiovascular diseases in humans.^39^, ^40^, ^41^, ^42^

The choice of concentration of folic acid used in this study with hypoxic human PAECs merits discussion. Humans depend on dietary folate and/or the synthetic form, folic acid, as a supplement to meet their biological needs because mammals lack the necessary enzymes to synthesize folic acid de novo.^43^ Human infants have basal plasma folic acid levels of approximately 0.1 μM and this is achieved from consumption of human milk or infant formula.^44^ Human milk contains folate acquired from the maternal diet and/or folic acid from maternal supplementation.^43^ Infant formulas contain folic acid at concentrations that mimic the concentration in human milk.^43^, ^44^, ^45^

For this study, we wanted to use a concentration of folic acid that would reflect plasma levels achieved in a human newborn if folic acid was provided as a supplement in addition to any folate or folic acid acquired from the diet. Additionally, we also wanted to use a concentration that would be considered to be safe in human infants and children and it is known that adverse effects have been associated with plasma folic acid levels approaching 0.7 μM in premature human infants.^46^, ^47^, ^48^ Therefore, we evaluated a folic acid concentration of 0.2 μM based on the desire to evaluate a concentration greater than basal levels (0.1 μM) but well below levels associated with adverse effects (0.7 μM).^46^, ^47^, ^48^

To our knowledge, we are the first to evaluate the effect of combining folic acid with l‐citrulline on NO signaling in PAECs from any species. Of importance, we found that although folic acid and l‐citrulline both independently reduced superoxide generation and increased the dimeric configuration of eNOS in hypoxic human PAECs; even greater reductions in superoxide production and elevations in eNOS dimer‐to‐monomer formation occurred when hypoxic human PAECs were co‐treated with folic acid and l‐citrulline. In other words, when concentrations of folic acid and l‐citrulline that should reflect safe plasma levels to target with in vivo therapy were used, co‐treatment with l‐citrulline and folic acid caused a greater improvement in eNOS dysfunction in hypoxic human PAECs than treatment with either alone.

It is also important to note that our finding that combined treatment with l‐citrulline and folic acid had a greater impact on eNOS re‐coupling than either alone suggests that l‐citrulline and folic acid improved eNOS recoupling by different and complementary mechanisms. For example, l‐citrulline can improve eNOS coupling by increasing the amount of bioavailable l‐arginine, which is needed for optimal eNOS coupling.^49^, ^50^ Folic acid can improve eNOS coupling by increasing bioavailability of BH_4_, which, in turn, promotes eNOS coupling by stabilizing eNOS in a dimeric configuration.^49^, ^51^ When eNOS is re‐coupled, reductions in superoxide generation occur concomitantly with increases in NO production. This is because in the homodimer or coupled state, electrons are transferred from the eNOS reductase domain to the oxygenase domain and NO is produced; whereas when eNOS becomes uncoupled, electrons are diverted to molecular oxygen‐producing superoxide instead of NO.^52^, ^53^ Therefore, our finding that combined therapy with l‐citrulline and folic acid had a greater impact on reducing superoxide production than did either therapy alone is not surprising. However, we had expected but did not find that NO production would increase in parallel with eNOS recoupling. Specifically, we had expected but did not find a greater increase in NO production in the presence of combined therapy with l‐citrulline and folic acid than with l‐citrulline alone. It is possible that co‐treatment with l‐citrulline and folic acid caused some yet to be described or understood negative effect on NO signaling that somewhat limited the ability to increase NO production despite the marked increase in eNOS recoupling. However, the ability to measure NO is known to be difficult and fraught with methodologic limitations.^54^ Thus, the relatively small number of samples studied and limitations with sensitivity of the assay are likely to have contributed to our inability to detect a difference in NO production between PAECs treated solely with l‐citrulline and those treated with the combination of l‐citrulline and folic acid.

Even without a synergistic effect on NO production, the more marked reduction in superoxide generation should result in greater beneficial physiological and therapeutic impacts with combined versus sole therapy. This is because it has been demonstrated in animal models of pulmonary hypertension that oxidative stress, including from superoxide, contributes to the development and pathogenesis of pulmonary hypertension.^53^, ^55^, ^56^, ^57^ In other words, it is important to recognize that reduced superoxide generation, even without a concomitant increase in NO production, is an important mechanism underlying the potential ability of both sole and combined treatments with l‐citrulline and/or folic acid to ameliorate pulmonary hypertension.

It is important to emphasize that we are aware that our findings in this study might have differed had we used other concentrations of folic acid and/or l‐citrulline. In fact, in a previous study using our chronically hypoxic piglet model, we did not find a greater impact on NO signaling from co‐treatment with folic acid and l‐citrulline than either alone.^38^ Of note, plasma folic acid levels averaged 0.8 μM in the folic acid‐treated piglets, both when treated solely with folic acid or co‐treated with l‐citrulline.^38^ It is quite possible that this plasma level of folic acid led to an increase in intracellular oxidative stress, as has been reported to occur in *Caenorhabditis elegans*.^58^ Supportive of this possibility, folic acid treatment failed to reduce superoxide generation in pulmonary arteries from the chronically hypoxic piglets.^38^ Indeed, although folic acid has been considered to be safe, there is growing awareness of the need for more research to assess the health effects of using folic acid in amounts exceeding current recommendations for upper limits.^59^, ^60^

Limitations of our study should be mentioned. We did not perform studies to evaluate the potential impact of sex on our findings. This is an important limitation worthy of future studies as there is an increasing amount of evidence supporting the influence of sex on the development of PH in adult humans. Another limitation is that we did not perform all of the mechanistic studies needed to thoroughly evaluate the full impact of l‐citrulline treatment alone or in combination with folic acid. In particular, additional studies assessing NO production, superoxide generation, and eNOS coupling in hypoxic human PAECs with escalating doses of l‐citrulline could be completed with and without the NOS inhibitor, l‐NAME, and these results compared with NO production in normoxic cells with and without l‐NAME to reveal differences in NO production that better correlate with changes in superoxide and eNOS coupling. Further, such studies could provide additional evidence describing the mechanisms involved in the synergistic effects of l‐citrulline and folic acid on eNOS coupling and function. In addition, because we were interested in mimicking the impact of l‐citrulline and folic acid in the presence of hypoxia‐associated disease, we did not perform studies in normoxic conditions. Performing studies under normoxic conditions is of future interest to help determine the impact of l‐citrulline and folic acid if used as a prophylactic treatment, that is, treatment started before the development of hypoxia‐associated pulmonary hypertension. Lastly, this study did not elucidate the precise threshold of l‐citrulline concentrations that increase NO bioavailability or induce arginase and future studies will be needed to identify the precise l‐citrulline concentrations that are safe and efficacious.

In summary, findings in this study are consistent with our previous findings and show that l‐citrulline can increase arginase II protein levels and arginase activity in hypoxic PAECs. None of the concentrations of l‐citrulline used in this or our previous study elevated arginase activity to levels that negated the ability of l‐citrulline to improve eNOS dysfunction in hypoxic PAECs. Nonetheless, the impact of l‐citrulline on arginase activity suggests that caution should be used when choosing doses of l‐citrulline to ameliorate PH in hypoxic conditions. Our findings suggest that doses of l‐citrulline that achieve plasma l‐citrulline levels ≤ 0.1 mM should not elevate arginase activity in PAECs of humans with PH due to hypoxic conditions. Moreover, our finding that combining l‐citrulline with folic acid had a greater impact on eNOS dysfunction than treatment with either alone has important therapeutic implications. Specifically, our findings serve as the impetus to explore the possibility that rather than using high‐dose monotherapies in future clinical trials, combining l‐citrulline and folic acid may offer enhanced therapeutic capacity and the potential to achieve efficacy at lower doses, minimizing potential adverse effects.

## AUTHOR CONTRIBUTIONS

**Matthew S. Douglass**: Data acquisition; analysis; interpretation; and article writing. **Mark R. Kaplowitz**: Data acquisition and analysis. **Yongmei Zhang**: Data acquisition and analysis. **Candice D. Fike**: Hypothesis generation; study design; data acquisition; analysis; interpretation; and article writing.

## CONFLICT OF INTEREST STATEMENT

Candice D. Fike is listed on a patent application for the use of lL‐citrulline‐citrulline as a therapeutic treatment for lung conditions.

## ETHICS STATEMENT

Institutional Review Board approval was not required as commerically available human pulmonary artery endothelial cells were used in this study.
